# Epidemiological and Clinical Characteristics of *Stenotrophomonas maltophilia* Isolates from Hospitalized Medical Patients; An Emerging Pathogen in the Non-Critically Ill

**DOI:** 10.3390/tropicalmed10090242

**Published:** 2025-08-28

**Authors:** Dimitrios Kouroupis, Charalampos Zarras, Maria Zarfeiadou, Christos Sanos, Elias Iosifidis, Chrysi Michailidou, Konstantina Mpani, Panagiotis Pateinakis, Theocharis Koufakis, Michail Doumas, Ioannis Goulis, Dimitrios Vlachakis, Athina Pyrpasopoulou

**Affiliations:** 1 2nd Propedeutic Department of Internal Medicine, Hippokration Hospital Thessaloniki, Konstantinoupoleos 49, 54642 Thessaloniki, Greece; dimcour841@gmail.com (D.K.); mariazarf@gmail.com (M.Z.); pateinakis@hotmail.com (P.P.); thkoyfak@auth.gr (T.K.); doumasm@auth.gr (M.D.); 2 Lab of Microbiology, Konstantinoupoleos 49, 54642 Thessaloniki, Greece; zarraschak6@gmail.com (C.Z.); chrysimicha_beni@yahoo.gr (C.M.); nantiabani@hotmail.com (K.M.); 3 3rd Department of Internal Medicine, Hippokration Hospital Thessaloniki, Konstantinoupoleos 49, 54642 Thessaloniki, Greece; sanos93christos@gmail.com (C.S.); igoulis@gmail.com (I.G.); vlachakisd@windowslive.com (D.V.); 4 Infectious Diseases Unit, Hippokration Hospital Thessaloniki, 54642 Thessaloniki, Greece; iosifidish@gmail.com

**Keywords:** *Stenotrophomonas maltophilia*, emerging pathogen, epidemiology, medical patients, outcome

## Abstract

Until recently, *Stenotrophomonas maltophilia* was considered a low-virulence pathogen, usually found as an environmental commensal and colonizer of moist abiotic surfaces. Lately, it has increasingly been implicated in invasive infections with high associated morbidity and mortality. Most epidemiological studies involving patients with *S. maltophilia* infections have recorded risk factors and their associations with outcomes in critically ill patients. The aim of this study was to investigate its epidemiology as a pathogen in patients hospitalized in medical wards and potential factors associated with mortality. For this purpose, *S. maltophilia*-positive cultures from patients admitted to medical wards from 1 January 2023 to 30 June 2025 were collected, demographics and patient characteristics were recorded and analyzed and associated with clinical outcome. Twenty-nine patients and their first positive *S. maltophilia* positive culture were included in the study with a direct attributable mortality of 27.6%. Patients with cardiovascular and chronic obstructive pulmonary disease more commonly developed respiratory tract infections. Among the recorded comorbidities, only diabetes was associated with worse outcome. Most of the strains retained sensitivity to co-trimoxazole and levofloxacin and treatment outcome was not affected by the choice of regimen. This study highlights the rise of *S. maltophilia* to a true pathogen affecting immunocompetent patients; in combination with its antimicrobial resistance, this justifies its recognition as an emerging pathogen of public health concern.

## 1. Introduction

*S. maltophilia* is a Gram-negative, non-fermenting rod which—until recently—was considered a commensal or opportunistic pathogen, mainly affecting immunocompromised, critically ill patients admitted to the ICU [1]. It is the single opportunistic pathogen of the genus *Stenotrophomonas* in humans [2]. Although most infections caused by *S. maltophilia* involve tracheobronchitis and blood stream infections, rarely, other affected sites have been reported, e.g., intrabdominal [3], skin [4], musculoskeletal, cardiac, or even eye infections [5].

*S. maltophilia* may be found in various environments; it is both an environmental bacterium occurring in water, rhizospheres, as well as a constituent of the animals’ microflora, and is also isolated from inanimate or moist surfaces, including plumbing systems [6]. It can contaminate and form biofilms on nosocomial surfaces and medical equipment which tend to retain moisture, such as endoscopes, nebulizers, or patients’ irrigation systems, leading in this way to healthcare-associated outbreaks [7,8]. Environmental hygiene measures help to reduce bacterial burden and contain spread within the nosocomial facilities [9]. Its tendency to colonize and cause systemic infections not only in the immunocompromised, but increasingly among the immunocompetent, has justifiably led to its recognition as a globally emerging pathogen [10].

Most *Stenotrophomonas*-associated infections are healthcare-associated. Community-acquired infections caused by this pathogen are rare, especially in immunocompetent patients. Overall prevalence of *S. maltophilia* among Gram-negative clinical isolates is low (2–3% of isolates implicated in bacteremia episodes [11,12]). The pathogen is significantly more frequently isolated from patients in the ICU, more so in the Respiratory ICU. In this setting, even though it can be the causative bacterial pathogen in a significant proportion of ventilator-associated pneumonias (VAP), it cannot be detected by standard molecular assays [13]. The latency in identification of the pathogen, its expected resistant phenotype to wide-spectrum antibiotics, including carbapenems and aminoglycosides, and patient-associated factors are all causes leading to significant mortality [14] despite the pathogen’s low virulence properties.

*S. maltophilia,* previously known as *Pseudomonas maltophilia* or *Xanthomonas maltophilia* is a motile, Gram-negative bacillus, slightly smaller in size than the other species in the *Stenotrophomonas* genus. It is aerobic, does not ferment glucose, and forms yellowish colonies on MacConkey agar [15]. *S. maltophilia* is catalase-positive but oxidase-negative, a feature which distinguishes this genus from the *Pseudomonas* one. It produces lipases, proteases and expresses β-lactamases. Pathogenicity of this microorganism is not elucidated. It can attach to abiotic structures, form biofilms, and from there migrate to the patients’ epithelia [16]. Additionally, *S. maltophilia* has the potency to become internalized in other monocellular organisms, which facilitates its survival in hospital water systems and enables reinfection of patients [17]. In general, colonization usually precedes the development of invasive infection. Risk factors for the progression to colonization and potentially infection appear to include previous administration of broad-spectrum antibiotics, prolonged hospitalization in departments associated with the prevalence of multidrug-resistant microorganisms and extensive use of antimicrobials, e.g., intensive care units, neoplasia with associated neutropenia and immunodeficiency, HIV infection, cystic fibrosis, presence of surgical wound, and mechanical ventilation [18].

The fact that *Stenophomonas* is not currently included in the spectrum of the widely applied molecular multi-pathogen detection assays, and the frequent co-isolation of other pathogens in the tested biological samples, makes timely diagnosis a challenge [2,19,20]. Besides the time-consuming standard microbiological cultures and susceptibility testing, MALDI-TOF MS (Matrix-Assisted Maser Desorption/Ionization Time-Of-Flight Mass Spectrometry) provides an excellent means of accurate pathogen identification [21]. Identifying the presence of *S. maltophilia* in a biological sample does not translate into active ongoing infection. Treatment of colonization has not been shown to lead to a better clinical outcome for the patients [22]. Careful clinical evaluation of the microbiological culture results and a proactive diagnostic approach are in order. As already mentioned, *S. maltophilia* is at large regarded as an opportunistic pathogen affecting mainly the immunocompromised; however, its increasing ability to cause infections in the immunocompetent justifies its characterization as a true pathogen under specific circumstances and highlights its potential as an emerging health challenge.

The aim of this study was to describe the epidemiological features of the patients admitted to medical wards from whom *S. maltophilia* was isolated, and to investigate associations between patient- and pathogen-related factors with the clinical outcome.

## 2. Materials and Methods

This was a single-center, retrospective, observational study conducted in Hippokration Hospital of Thessaloniki, an 800-bed capacity tertiary hospital in Northern Greece. The study included patients admitted to medical wards from 1 January 2023 to 30 June 2025 (30 months) with a positive culture for *S. maltophilia*. For this purpose, storage files of the Biomerieux Vitek 2 System were screened for all *S. maltophilia*-positive samples (blood, central venous catheter tips, sputum, bronchoalveolar lavage, ascitic fluid, bile, trauma and urine, cultured on MacConkey agar) within this time period. Repeated positive samples from the same patient were excluded. Sensitivities of the isolated pathogens for co-trimoxazole and levofloxacin were recorded and the strains were characterized as susceptible or non-susceptible according to the updated EUCAST susceptibility breakpoints. The patients’ medical records were retrieved and analyzed. Demographics (gender, age), admission and discharge date, co-morbidities, date of the positive culture, treatment and outcome were recorded. The colonization or infection was characterized as community-acquired if the patient’s history was negative for previous hospitalizations and the sample was taken within >48 h of admission, or healthcare-associated if the patient had been admitted to a hospital within the previous 3 months of this admission and/or the sample was taken after the first 48 h of the current admission. A note was made if more pathogens were cultured from the sample or the patient was diagnosed with another co-infection. Co-infection with other pathogens was defined as the presence of an organism other than *S. maltophilia* in the first positive clinically relevant biological sample for this pathogen. Patients were characterized as treated when targeted (appropriate/adequate/effective) treatment (levofloxacin, co-trimoxazole according to susceptibility testing) was initiated within 48 h after diagnosis of a positive microbiological sample [23].

Descriptive statistics were used for demographics (age, gender), positive sample type, types of comorbidities, presence of co-infections, duration of hospitalization prior to positivity for *S. maltophilia*, administration of treatment and outcome (directly related to the infection under examination or not). A univariate analysis was conducted to explore associations of variables with clinical outcome (30-day mortality). Categorical variables were analyzed using the Fisher’s exact test while continuous variables were assessed using parametric (Student’s *t*-test) or non-parametric (Mann–Whitney U test) methods as appropriate.

Analysis was performed with SPSS 20.0 with the chi-square and the independent samples *t*-test, as appropriate. The *p* value of <0.05 was considered statistically significant. The research adhered to the principles outlined in the Declaration of Helsinki and the protocol received approval from the local ethics committee (Approval No.: 31601/18-07-2025, FF:24-E-45).

## 3. Results

Twenty-nine patients (12 in 2023, 13 in 2024 and 4 during the first half of 2025) were included in the study. Seventeen patients (58.6%) were male. Mean age of the patients was 71.7 ± 3.3 years. Most of the patients tested positive in their sputum (14/29, 48.3%). *Stenotrophomonas*-related septicemia was recorded in 9/29 patients (31.0%). Less frequently, the pathogen was isolated from bronchoalveolar lavage, trauma or urine samples, respectively (Table 1). Among the 29 isolated strains, 4 (13,8%) were resistant to co-trimoxazole and 3 (10.3%) were resistant to levofloxacin. In 19/29 patients (65.5%) the colonization/infection was characterized as healthcare-associated. The most frequent co-morbidity was cardiovascular disease (19/29, 65.5%) followed by chronic obstructive pulmonary disease and immunosuppression (12/29, 41.4%). Less than half of the patients received treatment (14/29, 48.3%). Twelve people died during their hospitalization (41.4%); however, in only 8 patients (27.6% of cases) death was directly related to the pathogen. Overall, patients with different co-morbidities were not shown to be predisposed to specific types of infection. However, patients with cardiovascular disease (CVD), chronic obstructive pulmonary disease (COPD) and diabetes (DM) tended to develop respiratory tract infection more frequently than patients with other comorbidities, while the association of bacteremia was higher in patients with chronic neurological disorders (CND) (Table 2). No association was found between demographics, type of sample, and treatment with outcome related to all causes. Among patients with bacteremia, 2/9 did not receive treatment and both died, and from the 7 patients who received targeted antimicrobial treatment, 3/7 died (*p* = 0.151). Among existing co-morbidities, only diabetes mellitus was associated with worse outcome (Table 3).

## 4. Discussion

The nature and pathophysiology of *S. maltophilia* infections, an opportunistic pathogen rising to a true pathogen, are still not fully elucidated. It lingers in the nosocomial environment, forming biofilms on moist abiotic surfaces, and colonizes patients, especially those per definition prone to become colonized by multidrug-resistant bacteria. Most susceptible individuals are patients admitted to Intensive Care Units, undergoing surgical procedures, with central venous catheters inserted, and treated with broad-spectrum antibiotics, like carbapenems, to which *Stenotrophomonas* is intrinsically resistant [24]. Our study was designed to address the range and impact of isolation of *S. maltophilia* from medical, non-critically ill patients with various comorbidities and evaluate the morbidity and mortality associated with it. The results of our retrospective analysis indicate that *S. maltophilia* is identified as the aetiologic pathogenic microorganism also in infections recorded in non-immunocompromised, non-critically ill patients. In this context, *S. maltophilia* appears to be increasingly implicated in invasive systemic infections, more frequently bacteremia and respiratory tract infections but also in more atypical infections, such as infections of the musculoskeletal system, endophthalmitis/scleritis, or skin infections [25,26].

Mere colonization and even contamination of biological samples by reusable medical devices is difficult firmly exclude [27]. However, it is of importance a. to isolate the pathogen and determine its susceptibility profile and b. not to discard its presence as non-clinically significant or characterize the culture as false positive in cases of true infection [28]. The fact that infections involving *Stenotrophomonas* are often polymicrobial further complicates clinical management [10] but is not necessarily associated with more severe disease. Invasive infections are associated with significant mortality, both in the immunocompromised and in the immune-competent [1,29]. Several epidemiological studies have investigated the effect of patient- and pathogen/treatment-related factors on clinical outcome, mainly focusing on patients with associated bacteremia. Isolation of the pathogen from blood and bronchoalveolar lavage, but not from sputum, has been generally associated with higher mortality [30]. However, most of these studies included or exclusively addressed critically ill patients. In our study performed on patients hospitalized in medical wards, outcome was not associated with the site of infection.

Several studies have recorded a correlation of severe disease and death with advanced age, and not with specific comorbidities [31,32,33]. This association was even more prominent among ICU patients with a wide age variation. In our study, the only comorbidity that was significantly associated with worse outcome was diabetes mellitus, an association that was even stronger when deaths were restricted to those strictly associated with the pathogen. Inappropriate antibiotics in reported studies resulted in worse outcome; preferred targeted treatment (co-trimoxazole versus levofloxacin) was not associated with differences in outcome even in the critically ill, regardless of the site of infection [34]; meta-analysis favored monotherapy versus a combination regimen [35]. Our study did not reveal any association of outcome with the chosen appropriate regimen. The resistance rates found in our study are similar to those in published reports [36].

Our study is restricted by the relatively small number of patients, which does not allow for concrete statistical conclusions and does not firmly exclude the possibility of colonization with the pathogen, but for the case of bacteremia, especially in the case of polymicrobial cultures. However, it highlights the rise of *S. maltophilia* to a true pathogen, associated with significant mortality, affecting patients under not fully characterized circumstances, even immunocompetent patients.

## 5. Conclusions

Emerging pathogens are characterized as such by their ability to cause disease of increasing incidence, spread to hosts not previously affected by them and/or become more virulent or more easily transmitted. *S. maltophilia*, previously considered a commensal or at worst an opportunistic pathogen has established its position among clinically important Gram-negative pathogens. *S. maltophilia* is resistant to broad-spectrum antibiotics, e.g., carbapenems commonly used in septic patients, whose broad use may facilitate breakthrough infections. Moreover, it requires specialized targeted treatment, which needs to be initiated promptly to ensure a favorable clinical outcome. It becomes, therefore, obvious that its emerging isolation among microbiological specimens is rising to a public health concern even to immunocompetent, non-critically ill patients.

## Figures and Tables

**Table 1:** Demographics and epidemiological characteristics of medical patients with a positive culture sample for *Stenotrophomonas maltophilia* from 1.1.23 to 30.6.25.

**Parameter**	
Gender, male: n (%)	17 (58.6)
Age (years) (mean, SD)	71.7 (±3.3)
Healthcare associated and healthcare facility-onset (n, %)	19 (65.5)
**Positive sample type**	
Blood culture (n, %)	9 (31.0)
Sputum (n, %)	14 (48.3)
BAL (n, %)	3 (10.3)
Trauma (n, %)	3 (10.3)
Urine (n, %)	1 (3.4)
**Present comorbidity**	
COPD (n, %)	12 (41.4)
CVD (n, %)	19 (65.5)
Neoplasia (n, %)	7 (24.1)
Hematological disease (n, %)	3 (10.3)
Immunosuppression (n, %)	12 (41.4)
CKD (n, %)	2 (6.9)
DM (n, %)	7 (24.1)
CND (n, %)	8 (27.6)
Sensitivity to SXT (n, %)	25 (86.2)
Sensitivity to LVX (n, %)	26 (89.7)
Presence of co-infections (n, %)	16 (55.2)
Treatment (yes) (n, %) SXT (n, %) LVX (n, %)	14 (48.3) 4 (13.8) 10 (34.5)
Duration of admission prior to positive sample (days) (mean, SD)	7.9 (±1.7)
Duration of hospitalization (days) (mean, ±SD)	19.5 (±28.1)
Outcome; death (n, %)	12 (41.4)
Death related to S. maltophilia (n, %)	8 (27.6)

(N; number, SD; standard deviation, CVC; central venous catheter, BAL; bronchoalveolar lavage; COPD; chronic obstructive pulmonary disease, CVD; cardiovascular disease, CKD; chronic kidney disease; DM; diabetes mellitus; CND; chronic neurological disorder, SXT; co-trimoxazole, LVX; levofloxacin).

**Table 2:** Association of type of infection with co-morbidity.

Variable	Positive Sputum Sample (N = 14)	Negative Sputum Sample (N = 15)	*p* Value	Bacteremia (N = 9)	Non-Bacteremia (N = 20)	*p* Value
CVD, n (%)	11 (57.9%)	8 (42.1%)	0.153	5 (26.3%)	14 (73.7%)	0.449
COPD, n (%)	8 (66.7%)	4 (33.3%)	0.096	2 (16.7%)	10 (83.3%)	0.160
Neoplasia, n (%)	3 (42.9%)	4 (57.1%)	0.742	2 (28.6%)	5 (71.4%)	0.872
Hematological Disease, n (%)	1 (33.3%)	2 (66.7%)	0.584	1 (33.3%)	2 (66.7%)	0.928
Immune suppression, n (%)	5 (41.7%)	7 (58.3%)	0.550	3 (25.0%)	9 (75.0%)	0.555
CKD, n (%)	1 (50.0%)	1 (50.0%)	0.960	0 (0.0%)	2 (100.0%)	0.326
DM, n (%)	4 (57.1%)	3 (42.9%)	0.590	2 (28.6%)	5 (71.4%)	0.872
CND, n (%)	3 (37.5%)	5 (62.5%)	0.474	5 (62.5%)	3 (37.5%)	0.024

(N; number, CVD; cardiovascular disease, COPD; chronic obstructive pulmonary disease, CKD; chronic kidney disease; DM; diabetes mellitus; CND; chronic neurological disorder).

**Table 3:** Association of demographics, patient and pathogen-related parameters with outcome.

Variable	Death Due to All Causes, n = 12	Recovery n = 17	*p* [CI]	Death Related to *S. Maltophilia,* n = 8	Recovery n = 21	*p* [CI]
Gender (male; n, %)	6 (50.0)	11 (64.7)	0.428	4 (50.0)	13 (61.9)	0.561
Age (years; mean)	73.8	70.2	0.607 [−17.7–10.5]	79.5	68.7	0.151 [−25.9–4.2]
HAI (n, %)	9 (75.0)	10 (58.8)	0.367	6 (75.0)	13 (61.9)	0.507
**Type of sample**						
Blood culture (n, %)	5 (41.7)	4 (23.5)	0.298	3 (37.5)	6 (28.6)	0.642
Sputum (n, %)	6 (50.0)	8 (47.1)	0.876	4 (50.0)	10 (47.6)	0.909
BAL (n, %)	2 (16.7)	1 (5.9)	0.348	2 (25.0)	1 (4.8)	0.110
**Co-morbidity**						
COPD (n, %)	6 (50.0)	6 (35.3)	0.428	3 (37.5)	9 (42.9)	0.793
CVD (n, %)	8 (66.7)	11 (64.7)	0.913	5 (62.5)	14 (66.7)	0.833
Neoplasia (n, %)	2 (16.7)	5 (29.4)	0.430	0 (0.0)	7 (33.3)	0.061
Hematological disease (n, %)	1 (8.3)	2 (11.8)	0.765	1 (12.5)	2 (9.5)	0.814
Immunosuppression (n, %)	4 (33.3)	8 (47.1)	0.460	2 (25.0)	10 (47.6)	0.269
CKD (n/total, %)	0 (0.0)	2 (11.8)	0.218	0 (0.0)	2 (9.5)	0.366
DM (n, %)	5 (41.7)	2 (11.8)	0.064	4 (50.0)	3 (14.3)	0.045
CND (n, %)	4 (33.3)	4 (23.5)	0.561	3 (37.5)	5 (23.8)	0.461
Sensitivity to SXT (n, %)	10 (83.3)	15 (88.2)	0.706	6 (75.0)	19 (90.5)	0.280
Sensitivity to LVX (n, %)	10 (83.3)	16 (94.1)	0.348	7 (87.5)	19 (90.5)	0.814
Presence of co-infections (n/total, %)	6 (50.0)	10 (58.8)	0.638	5 (62.5)	11 (52.4)	0.624
Treatment (yes) (n, %) SXT (n, % of treated) LVX (n, % of treated)	8 (66.7.3) 3 (37.5) 5 (62.5)	12 (70.6) 2 (16.7) 10 (83.3)	0.494 0.173	5 (62.5)	9 (42.9)	0.344
Treatment in patients with bacteremia (yes) (n/total, %)	3/7 (42.9)	4/7 (57.3)	0.151	2/3 (66.7)	1/3 (33.3)	N/A
Duration of admission prior to positive sample (days) (mean, SD)	10.3	6.2	0.251 [−11.2–3.1]	7.3	8.1	0.831 [−7.2–8.9]
Duration of hospitalization (days) (mean, ±SD)	29.5	12.4	0.108 [−38.2–4.0]	18.6	19.8	0.921 [−23.2–25.6]

(N; number, SD; standard deviation, CVC; central venous catheter, BAL; bronchoalveolar lavage; COPD; chronic obstructive pulmonary disease, CVD; cardiovascular disease, CKD; chronic kidney disease; DM; diabetes mellitus; CND; chronic neurological disorder, SXT; co-trimoxazole, LVX; levofloxacin, N/A; not applicable).

## Data Availability

The data presented in this study are available on request from the corresponding author due to privacy issues.
